# Host Suitability of a Gregarious Parasitoid on Beetle Hosts: Flexibility between Fitness of Adult and Offspring

**DOI:** 10.1371/journal.pone.0018563

**Published:** 2011-04-19

**Authors:** Li Li, Jianghua Sun

**Affiliations:** 1 State Key Laboratory of Integrated Management of Pest Insects and Rodents, Institute of Zoology, Chinese Academy of Sciences, Beijing, China; 2 School of Life Sciences, Guizhou Normal University, Guiyang, China; Ghent University, Belgium

## Abstract

Behavioral tactics play a crucial role in the evolution of species and are likely to be found in host-parasitoid interactions where host quality may differ between host developmental stages. We investigated foraging decisions, parasitism and related fitness in a gregarious ectoparasitoid, *Sclerodermus harmandi* in relation to two distinct host developmental stages: larvae and pupae. Two colonies of parasitoids were reared on larvae of *Monochamus alternatus* and *Saperda populnea* (Cerambycidae: Lamiinae). Paired-choice and non-choice experiments were used to evaluate the preference and performance of *S. harmandi* on larvae and pupae of the two species. Foraging decisions and offspring fitness-related consequences of *S. harmandi* led to the selection of the most profitable host stage for parasitoid development. Adult females from the two colonies oviposited more quickly on pupae as compared to larvae of *M. alternatus*. Subsequently, their offspring development time was faster and they gained higher body weight on the pupal hosts. This study demonstrates optimal foraging of intraspecific détente that can occur during host-parasitoid interactions, of which the quality of the parasitism (highest fitness benefit and profitability) is related to the host developmental stage utilized. We conclude that *S*. *harmandi* is able to perfectly discriminate among host species or stages in a manner that maximizes its offspring fitness. The results indicated that foraging potential of adults may not be driven by its maternal effects, also induced flexibly with encountering prior host quality.

## Introduction

All insect parasitoids face the problem of finding sufficient high-quality resources for growth, maintenance and reproduction. When foraging in a heterogeneous environment, parasitoids often encounter a variety of host species at different developmental stages. These hosts may differ in body size, behavioral defense, physiological and immunological status [1]–[3]. Optimal foraging strategy in host selection decisions by parasitoids may be determined by changes in host resources or quality [4]–[5].

In the context of host selection, parasitoids that forage optimally should adopt behaviors that provide the highest fitness return or profitability in relation to the host size or age availability distribution [4]–[5]. Many parasitoids are able to assess the quality of hosts through host size and selectively parasitize hosts of a certain size [6]–[7]. Host stage-selective feeding and oviposition reduce competition for hosts between adult female and her progeny or among progeny, with a corresponding increment in offspring survival and performance [8]. One of the optimal patterns to emerge from previous studies on the life history strategies of parasitoids is that large body size confers greater fitness [6] and closely correlates with the stage of the host at parasitism [9]–[10].

For gregarious idiobiont parasitoids, which often kill or paralyze the attacked hosts, the amount of resource may be an available cue for adult and progeny fitness [11]. Mackauer and Sequeira (1993) proposed that parasitoids develop in a closed resource system, and should always grow at the maximum possible rate to take advantage of a diminishing food supply [12]. Parasitoids are generally expected to attack larger or near mature hosts, which contain a greater quantity of resources than small or juvenile hosts. Progeny that emerge from larger hosts likely benefit from larger adult size that tends to be positively correlated to fitness parameters, such as fecundity and survival of parasitoids [5], [12]. Selection of the most profitable host stage also influences sex allocation patterns in arrhenotokous parasitoids [6]. A higher proportion of females may be produced from larger hosts because of the greater nutritional requirement and reproductive benefits for the female progeny [9].

*Sclerodermus harmandi* Bursson (Hymenoptera: Bethylidae) is a successful natural parasite of *Monochamus alternatus* Hope (Coleoptera: Cerambycidae), the most important vector of the pinewood nematode, *Bursaphelenchus xylophilus* Steiner et Buhrer in Japan and China [13]–[14]. *S. harmandi* is a synovigenic anautogenous species in which oogenesis takes place after females feed on hosts and is stimulated by direct access to suitable hosts for oviposition [8], [15]. Because host meals are essential for oogenesis throughout the reproductive lifetime, parasitoids are often considered to have inherent parental conflicts of interest vis-à-vis their progeny [16]–[17]. *S. harmandi* females permanently paralyze larvae and pupae of hosts prior to feeding and oviposition. Parental females remain with broods until completion of offspring development while young females disperse after mating to search for cerambycid larval and pupal chambers inside infested trees. Lastly, *S. harmandi* likely uses effective searching tactics in finding their hosts which tend to be solitary wood-boring insects in cryptic situations (trunk, wood and seed) [18]–[20].

We report on the responses of *S. harmandi* to various host stages, and subsequently, the suitability of hosts for parasitoid fitness-related performance. Trade-offs related to the maternal host in different developmental stages is tested by using two parasitoids populations. First we hypothesize that *S. harmandi* can adaptively integrate foraging preference with adult and offspring performance as it relates to host development stages. Under laboratory conditions, we tested the prediction by measuring feeding preference of *S. harmandi* females from two colonies using two host species in different stages. We further investigated the effects of host stages on host preference and performance of *S. harmandi*. The relative suitability of larvae and pupae of two host species for *S. harmandi* was determined by measuring performance of parental parasitoid (pre-oviposition period and fecundity) and progeny (developmental time, body weight, survival and sex ratio).

## Results

### Behavioral response to host stage

**Figure 1:**
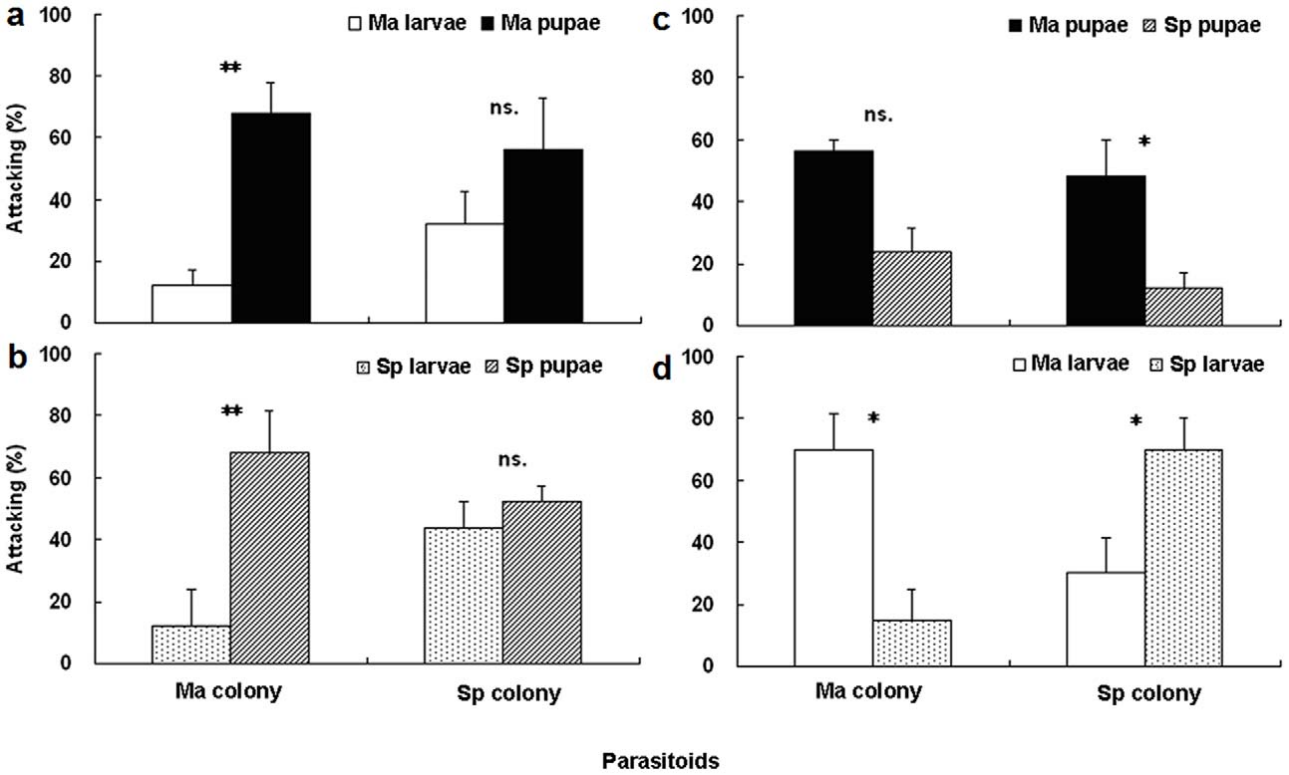
Feeding preference of *Sclerodermus harmandi* from different culturing systems on two development stages of hosts: (1a) pupae vs. larvae of Ma, (1b) pupae vs. larvae of Sp, (1c) pupae of Ma vs. Sp, and (1d) larvae of Ma vs. Sp. Abbreviations used: Ma, *Monochamus alternatus*; Sp, *Saperda populnea*; Ma larvae, Ma pupae, larvae and pupae of *M*. *alternatus*, respectively; Sp larvae, Sp pupae, larvae and pupae of *S*. *populnea*, respectively (Mean±SE, *n* = 25 females in each treatment, ** P<0.01, * P<0.05 and ns. P>0.05).

When the host stages were exposed simultaneously to *S. harmandi* females from *M. alternatus* and *S. populnea* colonies (Ma colony and Sp colony), significant preferences were detected in the selection of two stages (Fig. 1). A higher proportion of Ma colony landed on pupae than on larvae in 24 h (Fig. 1a,b; χ^2^_1_ = 9.800, P<0.001; χ^2^_1_ = 9.800, P<0.001). However, females of Sp colony had no significant differences between larvae and pupae of *M. alternatus* or *S. populnea* in 24 h (Fig. 1a,b; χ^2^_1_ = 1.636, P = 0.201; χ^2^_1_ = 0.167, P = 0.683).

Sp parasitoids preferred to feed on pupae of *M. alternatus* than those of *S. populnea* in 24 h (Fig. 1c; χ^2^_1_ = 5.400, P<0.05). For the Ma colony, no significant differences were detected between pupae of *M. alternatus* and *S. populnea* in 24 h (Fig. 1c; χ^2^_1_ = 3.200, P = 0.074). Between pupae and larvae of *S. populnea*, the Ma colony preferred to attack pupae than larvae of *S. populnea* in 24 h (Fig. 1c; χ^2^_1_ = 9.800, P<0.005) which the Sp colony had no preferences between them (Fig. 1c; χ^2^_1_ = 0.167, P = 0.683). Females from the two colonies showed significant preference to their original hosts (Fig. 1d; Ma colony, χ^2^_1_ = 8.909, P<0.005; Sp colony, χ^2^_1_ = 3.846, P<0.05).

### Adult performance to host stage

**Figure 2:**
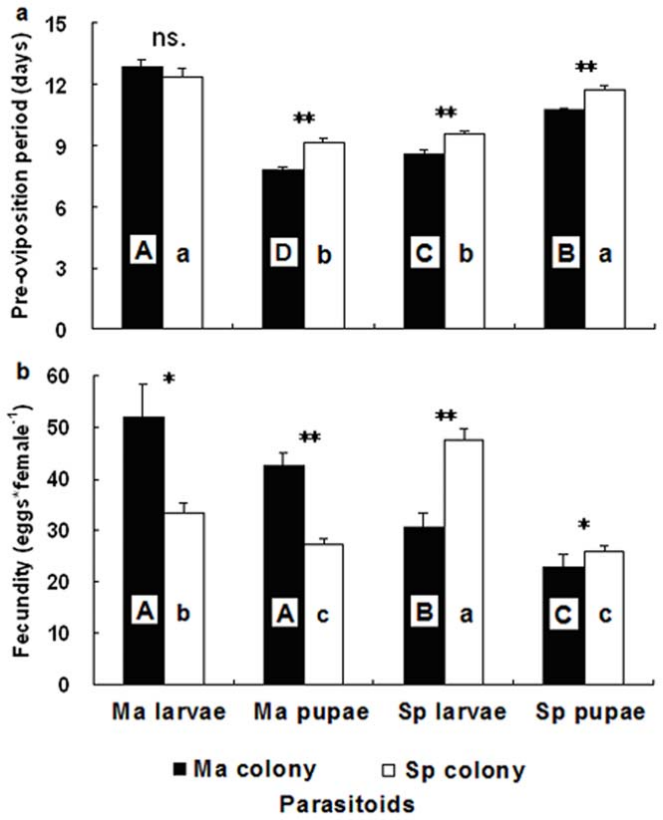
The performance of *Sclerodermus harmandi* females from different culturing systems on two host species and stages: (2a) pre-oviposition period of adult females, (2b) female fecundity. Same alphabets on columns indicate no significant differences (P<0.05). For abbreviations see Figure 1 (Mean±SE, *n* = 20–24 females in each treatment, ** P<0.01, * P<0.05 and ns. P>0.05).

Adult performance of females varied significantly between host species and host stages. When the two host species and stages were exposed separately to females, the shortest periods of pre-oviposition by Ma colony females were observed in the parasitism of *M. alternatus* pupae (Fig. 2; ANOVA; *F*_7,154_ = 50.93, P<0.001). However, pre-oviposition periods of Ma colony females were significantly prolonged by 2.1 days on pupae as compared to larvae of *S. populnea* (Fig. 2; *t*-test: *t*_38_ = 8.404, P<0.001). The pre-oviposition periods of Sp colony females were clearly longer on larvae of *M. alternatus* than on larvae of *S. populnea* (Fig. 2; *t*-test: *t*_40_ = 8.457, P<0.001) and pupae of *M. alternatus* (Fig. 2; *t*-test: *t*_39_ = −7.484, P<0.001).

In the no-choice tests, female fecundity of Ma colony was higher on larvae and pupae of *M. alternatus* than on the other host *S. populnea*, and they were not significantly different to each other (Fig. 2; *t*-test: *t*_36_ = −1.344, P = 0.187). Females of Sp colony laid more eggs on larvae than pupae of *S. populnea* (Fig. 2; *t*-test: *t*_33_ = −8.88, P<0.001). However, when it was reared for only one generation on larvae and pupae of the other host *M. alternatus*, fecundity of Sp colony showed no differences between larvae and pupae of *M. alternatus* (Fig. 2; *t*-test: *t*_36_ = −1.880, P = 0.07), but lower than on each stage of *S. populnea* (Fig. 2).

### Offspring performance response to host stage

**Figure 3:**
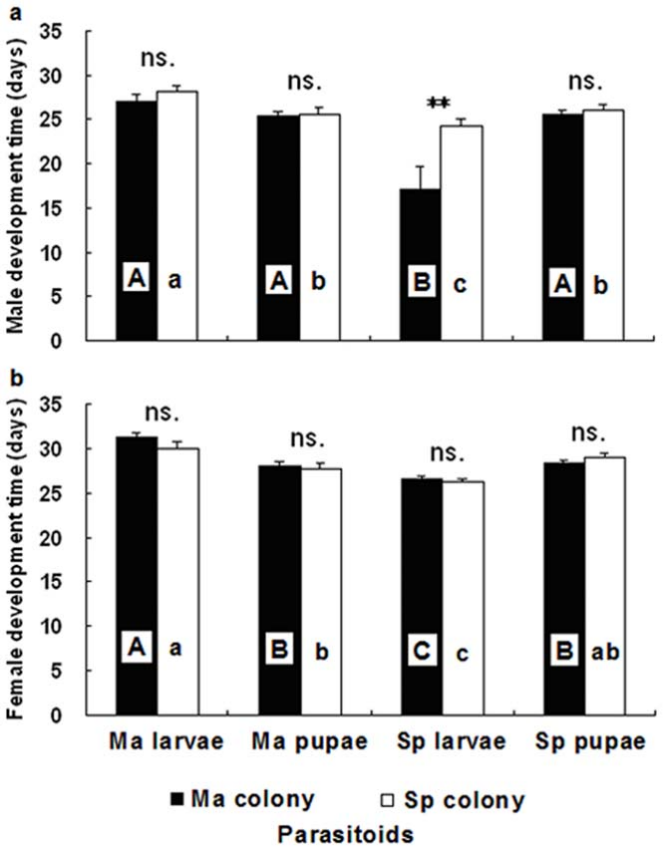
Offspring development time of *Sclerodermus harmandi* from different culturing systems on two host species and stages: (3a) female offspring, (3b) male offspring. Same alphabets on columns indicate no significant differences (P<0.05). For abbreviations see Figure 1 (Mean±SE, *n* = 20–24 females in each treatment, ** P<0.01 and ns. P>0.05).

Female offspring of Ma colony always had shorter development time on larvae of *S. populnea* than on the other host species and stages (Fig. 3; ANOVA; *F*_7,131_ = 14.76, P<0.001). Likewise, male offspring of Ma and Sp colony had shorter development time on larvae of *S. populnea* than on the other host species and stages (Fig. 3; ANOVA; *F*_7,125_ = 7.81, P<0.001). Moreover, the male offspring development time of Sp colony was shorter than Ma colony (Fig. 3; *t*-test: *t*_38_ = −2.796, P<0.01).

**Figure 4:**
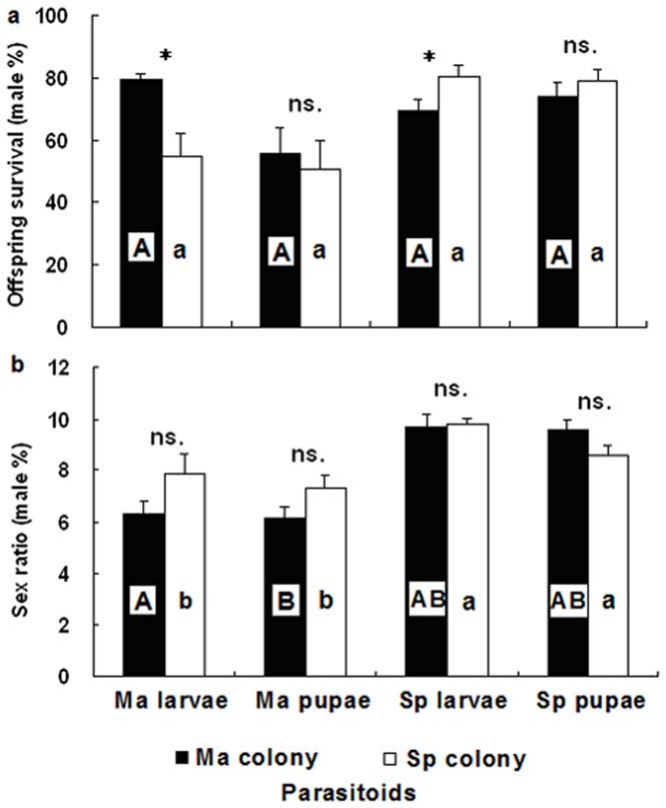
Offspring survival and sex ratio of *Sclerodermus harmandi* from different culturing systems on two host species and stages: (4a) offspring survival, (4b) sex ratio (proportion of males). Same alphabets on columns indicate no significant differences (P<0.05). For abbreviations see Figure 1 (Mean±SE, *n* = 20–24 females in each treatment, * P<0.05 and ns. P>0.05).

The Ma colony had higher offspring survival on larvae than pupae of *M. alternatus* (Fig. 4; *t*-test: *t*_33_ = 2.756, P<0.01), whereas there was no significant differences between larvae and pupae of *S. populnea* (Fig. 4; *t*-test: *t*_33_ = 0.854, P = 0.399). However, lower offspring survival was observed in the Sp colony on larvae pupae of *M. alternatus*, and they were not significantly different to each other (Fig. 4; *t*-test: *t*_25_ = 1.391, P = 0.176). Offspring sex ratio of the two colonies were female-biased and did not differ between the two host species and host stages (Fig. 4; ANOVA; *F*_7,130_ = 1.85, P = 0.08).

**Figure 5:**
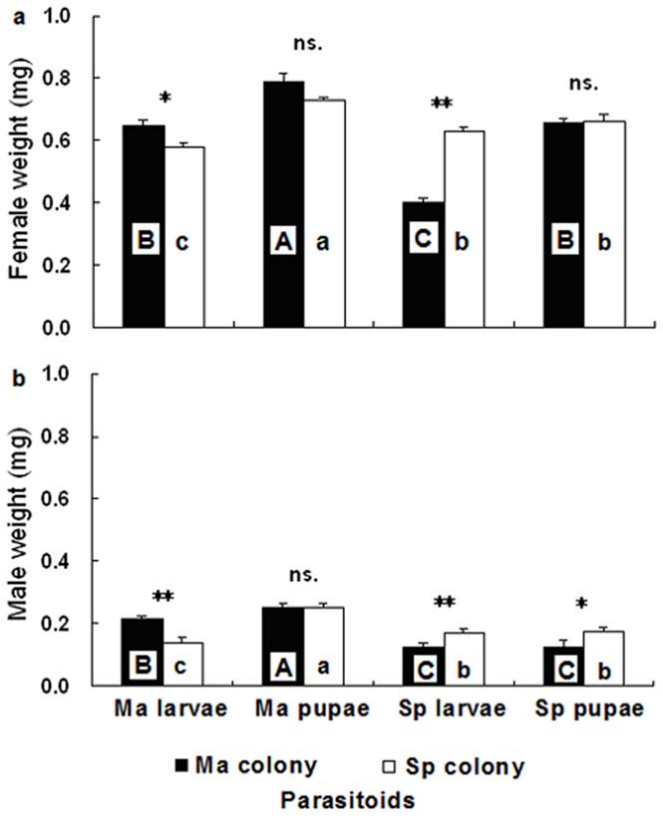
Offspring weight of *Sclerodermus harmandi* from different culturing systems on two development stages of hosts: (5a) adult females, (5b) adult males. Same alphabets on columns indicate no significant differences (P<0.05). For abbreviations see Figure 1 (Mean±SE, *n* = 300 females, 200 males in each treatment, ** P<0.01, * P<0.05 and ns. P>0.05).

Adult offspring of Ma and Sp colony that developed on pupae of *M. alternatus* had the highest weight compared to other host species and stages (Fig. 5a, b; ANOVA; Ma colony, female, *F*_3,116_ = 58.995, P<0.001; male, *F*_3,116_ = 21.209, P<0.001; Sp colony, female, *F*_3,116_ = 50.553, P<0.001; male, *F*_3,116_ = 21.169, P<0.001). However, compared to the weight of offspring that developed on pupae of *M. alternatus*, there were differences between Ma and Sp colony (Fig. 5a, b; *t*-test: female, *t*_58_ = 1.904, P = 0.062; male, *t*_58_ = 0.199, P = 0.844). Offspring weight of Ma colony reared on larvae of *M. alternatus* was significantly higher than on those of Sp colony (Fig. 5a, b; *t*-test: female, *t*_58_ = 2.586, P<0.05; male, *t*_58_ = 4.849, P<0.001). Surprisingly, after switching brood hosts of Ma colony on larvae of *S. populnea*, the body weight of its female offspring was lower than on pupae of the host (Fig. 5b; *t*-test: *t*_58_ = −11.811, P<0.001), whereas there were no differences in the body weight of its male offspring (Fig. 5b; *t*-test: *t*_58_ = −0.177, P = 0.861). Adult offspring of Sp colony had higher weight on larvae than pupae of *M. alternatus* (Fig. 5a, b; *t*-test: female, *t*_58_ = −9.352, P<0.001; male, *t*_58_ = −8.195, P<0.001). The body weight of adult offspring of Sp colony were not siginificantly different between larvae and pupae of *S. populnea* (Fig. 5a, b; *t*-test: female, *t*_58_ = −1.590, P = 0.117; male, *t*_58_ = −0.264, P = 0.793).

## Discussion

Parasitoid wasps frequently have been used as model organisms for the study of life history evolution [5], [21]–[23]. The preference, utilization and performance of *S. harmandi* on larvae and pupae of *M. alternatus* and *S. populnea* were used to ascertain whether female host selection and subsequent fitness corresponded with host quality. *S. harmandi* females from the two colonies (Ma colony and Sp colony) oviposited more quickly on pupae than on larvae of *M. alternatus*. Subsequently, their offspring development time was faster and they gained higher body weight on the pupal hosts. The capacity of *S. harmandi* females to grow on hosts of two developmental stages suggests an ability to adjust to the constraints specific to each age or stage.

Many studies have sought to establish if parasitoid host selection conforms to predictions of ‘optimal foraging theory’ [24]–[25] and ‘optimal oviposition theory’ [9], [26]. Both theories stress that there were trade-offs between maternal fitness through host-feeding [27]–[28] and enhanced reproduction through oviposition on the same host individual as used for host-feeding [9], [29]. However, we found that optimum foraging strategy was combined with optimum oviposition strategy. Because the selection of the most profitable host stage for parasitoid development was due to foraging decisions and offspring fitness-related consequences of *S. harmandi*,this may not mean that one of offspring's success was built on the side of the expense of maternal fitness.

This study demonstrates the existence of a flexible foraging strategy that occurs during host-parasitoid interactions, of which the quality of the parasitism (highest fitness benefit and profitability) is related to the host developmental stage utilized. The better quality (higher fitness benefit and profitability) of pupae compared to larvae of *M. alternatus*, resulted in more pupae being successfully parasitized, shorter pre-oviposition time by adult parasitoids and higher body weight gained by their offspring. However, *S. harmandi* females would probably use suboptimal hosts when their availability, either in terms of accessibility or abundance, makes them profitable even though individuals using these resources may suffer in their individual fitness. This study clearly stressed the need to incorporate the diversity of optimal foraging strategy existing between parasitoids and host life-history traits (physiological and behavioural characteristics) in the prediction of the host-parasitoid interactions. In addition, female *S. harmandi* from both colonies demonstrated significantly higher preference on maternal host larvae. These results were consistent with our previous works as the preference is likely to be an adaptation to a specific host over multiple generations [15], [30].

Additionally, the effect of maternal host/colony is not replicated as all parasitoid individuals come from the same long-term colonies. Any differences found between the two colonies is not necessarily due to the maternal host, but could be caused by other reasons. There are some behavioural defenses between larvae and pupae of *Monochamus alternatus*. The latter is more active and the former is more defensive. For most parasitoid wasps, successful parasitism is associated with multiple trade-offs between different physiological and behavioural constraints. Indeed, host size varied with host development stage generally determines host's physiological and behavioural defense, particularly where juvenile parasitoids consume virtually all host tissues before pupation [5], [10], [28], [31]. The immature beetles respond to the parasitoid attacks by shaking their body and biting the attackers. These defense behaviors have been shown to differ between immature host stages as pupae move far less than larvae and lack the functional mandibles to defend themselves.

On the other hand, the host's immune system, metabolism, and nutritional status changes with development and can influence the quality of immature hosts, and thus, result in a lower fitness. We speculated that it would result from a trade-off of *S. harmandi* females to immune response of suboptimal hosts or non-maternal hosts. Moreover, the hosts' behavioral and immune system could explain its longer pre-oviposition time of *S. harmandi* on those hosts with bigger bodies and higher activity.

The success of mass rearing of *S. harmandi* depended on its efficient food consumption. Our experiments focused on how trade-offs related to the maternal host can be affected by host species and its developmental stages. However, there are still some challenges to improve *S. harmandi*'s mass-rearing efficiency in the laboratory and its parasitism to target-hosts in the field. Future work should pay more attention to investigate the effects of host's immune system, metabolism and nutritional status on the behavioral and physiological conditioning of *S. harmandi*.

Host selection decision in parasitoids depends on the quality of the hosts, but also on their availability in the habitat. Our experimental treatments omitted much of the complexity of parasitoid natural population and host species in the inconstant field. For example, the host suitability experiments were limited to two host species and two developmental periods. Under natural conditions, choice of the targeted host developmental stage in the field, regulation of parasitoid numbers released, and introduction of food supplements are operational factors with a potential to influence the level of biological control.

## Materials and Methods

### Experimental insects

The effects of host stage were examined by conducting the following two experiments: 1) feeding choices by female *S. harmandi* on larvae and pupae; and 2) fitness-related performance by adult females and offspring on larvae and pupae. Base stocks of *S. harmandi* for all experiments were obtained from two laboratory colonies. Single-host colony of *S. harmandi* was maintained separately on the larvae of two different hosts for ten successive generations. One colony was reared solely on the larvae of *M. alternatus* (Ma colony) whereas the other colony was reared solely on the larvae of *Saperda populnea* (Sp colony). *S. populnea* (Cerambycidae: Lamiinae) is commonly used as a substitute host in mass rearing of *S. harmandi*. Final instar larvae and pupae of *M. alternatus* were collected from Zhejiang province whereas larvae and pupae of *S. populnea* were provided by the Xishan Forest Factory. All larvae and pupae of two species were stored at 8∼10°C prior to use in parasitoid rearing.

For both colonies, individual *S. harmandi* were reared in vials (7.5 cm in height×1.2 cm in diameter), each blocked with a cotton plug on the port and kept at 25±5°C, 70% RH under a LD 14∶10 h. Mated female *S. harmandi* were fed on 10% honey for 5–6 days and then presented with host larvae in each vial for subsequent ovipositioning/feeding. For the colony reared on *M. alternatus* the larvae were presented at a ratio of 3∶1 parasitoid/host, whereas a ratio of 1∶1 parasitoid/host was used for those reared on *S. populnea*. The difference in ratios was due to the differences in larval size [20]. Generation times for *S. harmandi* were approximately 35 days on *M. alternatus* and 25 days on *S. populnea*.

### Host stage preference experiments

To test the effect of host stage on subsequent host choices by females (Experiment 1), we used the two types of *S. harmandi* (Ma colony and Sp colony), based on their rearing histories. A two-choice test was used to determine feeding preference of the two treatment groups. Larvae and pupae of *M. alternates* and *S. populnea* were used as testing hosts. There were pairwise comparison between the four species conducted as follows: 1) larvae vs. pupae of *M. alternates* (Ma larvae vs. Ma pupae); 2) pupae of *M. alternates* vs. *S. populnea* (Ma pupae vs. Sp pupae); 3) larvae vs. pupae of *S. populnea* (Sp pupae vs. Sp pupae); and 4) larvae of *M. alternates* vs. larvae of *S. populnea* (Ma larvae vs. Sp larvae). In a two-choice bioassay, the following choices were offered simultaneously in a glass Petri dish of 12 cm in diameter: a) one larva and one pupa of the same species; b) two larvae or pupae (one of each species) One female wasp was put in the center of each dish with a fine brush. For each of the two colonies, five females per dish were tested simultaneously with five replicates (n = 25 females). Choice tests were conducted at 25–26°C under a lamp (100 Lx) hanging approximately 0.5 m above the roof of the experimental arena.

Feeding preference was expressed as the successful host-selecting rate (SSR) of female *S. harmandi* to two hosts at 24 h. In a bioassay with successful host selection, female *S. harmandi* walk, search and probe throughout the arena, and generally do not change positions for 24 h after making their selection [30]. SSR of *S. harmandi* was defined as the proportion of females that attacked hosts with simultaneously probing, stinging and feeding behaviors for at least 5 min [SSR =  (number of females attacking on each host / total number of females) * 100%] [20], [30].

### Host stage suitability experiments

In Experiments 2, we determined the effects of two developmental stages of hosts on performance of female *S. harmandi* and offspring, respectively. We used one hundred and sixty *S. harmandi* of each of Ma colony and Sp colony, as used in Experiment 1. Mated female *S. harmandi* were fed on 10% honey for 5–6 days and then kept at 8–10°C. Females of each treatment group were placed at room temperature at least for 1 h before testing and used only once. No-choice tests were carried out in a glass vial (as mentioned above) and tested at 25–26°C and 14∶10 h light∶dark photoperiod regime.

In the no-choice bioassay, a host was offered to the mated females, at a ratio of 3∶1 and 1∶1 for larvae of *M. alternatus* and *S. populnea*, respectively. The same experiments were performed for pupae of *M. alternatus* and *S. populnea*, respectively. Each treatment was replicated twenty times (n = 320 females). Adult fitness consequences on each host were recorded after the female wasps oviposited and completed offspring development on the paralyzed hosts for 30–40 days [19]. Female fecundity (number of eggs laid per female) and pre-oviposition period (days) on two development stages of the hosts were observed and recorded. The pre-oviposition periods of adult females were counted as time from emergence to first reproduction in females (APOP) [18]. Host mortality was checked daily and dead hosts were replaced.

In Experiments 3, offspring performance was determined by no-choice experiments. The larvae and pupae of *M. alternatus* were presented at a ratio of 3∶1 parasitoid/host whereas a ratio of 3∶4 parasitoid/host was used for those of *S. populnea* due to differences in larval and pupal weight between the two species. The mean weight of one *M. alternatus* larva or pupa is equal to four *S. populnea* larvae [20]. Females of the two colonies were allowed to oviposit on larvae and pupae of *M. alternatus*, respectively. The same experiments were performed on *S. populnea*. Twenty eggs newly laid by females were left on their original hosts and offspring performance was determined by parameters of total development time (days), weight of eclosing adult (mg), survival (%) and sex ratio (proportion of male). Each treatment was replicated twenty times (a total of 400 eggs). Host mortality was checked daily and dead hosts were replaced.

### Data analysis and statistics

Statistical analyses for this study were performed using SPSS 13.0 for Windows (SPSS Inc., Chicago, IL, USA). Chi-square test was used to compare feeding preference (successful host-selecting rate) to two host species among parasitoids, and to larvae vs. pupae of each species. One-way analysis of variance (ANOVA) and Student-Newman-Keuls (S-N-K) multiple comparisons test (p<0.05) were performed to assess the differences in adult performance (pre-oviposition period and realized fecundity) and offspring performance (mean development time, body weight, survival and sex ratio) of *S. harmandi* from two colonies. Differences in these fitness measures between host stage and host species were analyzed with independent sample *t*-test. Pre-oviposition period, mean development time and body weight were transformed by a square root transformation prior to the analysis. The percentage-based data (survival and sex ratio) were analyzed following an arcsine square root transformation.
